# Bioactive Compound Content and Cytotoxic Effect on Human Cancer Cells of Fresh and Processed Yellow Tomatoes

**DOI:** 10.3390/molecules21010033

**Published:** 2015-12-25

**Authors:** Assunta Raiola, Rita Del Giudice, Daria Maria Monti, Gian Carlo Tenore, Amalia Barone, Maria Manuela Rigano

**Affiliations:** 1 Department of Agricultural Sciences, University of Naples Federico II, Via Università 100, 80055 Portici (Naples), Italy; assuntaraiola@hotmail.com (A.R.); mrigano@unina.it (M.M.R.); 2 Department of Chemical Sciences, University of Naples Federico II, Complesso Universitario Monte Sant’Angelo, via Cinthia 4, 80126 Naples, Italy; rita.delgiudice@unina.it (R.D.G.); mdmonti@unina.it (D.M.M.); 3 Department of Pharmacy, University of Naples Federico II, Via D. Montesano 49, 80131 Naples, Italy; giancarlo.tenore@unina.it

**Keywords:** *Solanum lycopersicum*, phenolic compounds, yellow fruit, fresh fruit, processed fruit, cytotoxicity

## Abstract

Tomato, as a fresh or processed product, has a high nutritional value due to its content of bioactive components such as phenolic compounds. Few studies describe the effect of processing on antioxidant content and the cancer cell growth inhibition activity. In this study we determined the phenolic and ascorbic acid content of three yellow tomato varieties, before and after thermal processing. Moreover, we determined the antioxidative power and tested the effects of tomato extracts on three human cancer cell lines. We found that the amount of phenolic acids (chlorogenic acid and caffeic acid) decreased in all the samples after processing, whereas the flavonoid content increased after the heat treatment in two samples. A cytotoxic effect of tomato extracts was observed only after processing. This result well correlates with the flavonoid content after processing and clearly indicates that processed yellow tomatoes have a high content of bioactive compounds endowed with cytotoxicity towards cancer cells, thus opening the way to obtain tomato-based functional foods.

## 1. Introduction

Among crops, tomato (*Solanum lycopersicum*), with a total production of around 160 million tons per year, is the second most important source of nourishment (after potatoes) for the World’s population [1]. Its consumption has increased in the last few years with the commercialization of several processed products such as sauces, juices, soups and purees. It has been estimated that 35% of raw tomatoes are consumed as sauces, 18% as tomato paste, 17% as canned tomatoes, 15% are transformed into juices, and 15% into catsup [2,3].

Tomato fruits show a high nutritional value, due to their content of several important micronutrients such as carotenoids, vitamins (C and E) and phenolic compounds [4,5]. Recent studies have demonstrated that the regular intake of tomato, either fresh or processed, is associated with a reduced risk of inflammation, cancer, cardiovascular diseases, diabetes and obesity and can increase cell protection from DNA damage by oxidant species [3,6]. In particular, phenolic compounds, including phenolic acids (chlorogenic, caffeic, ferulic, gallic, *p*-hydroxybenzoic, protocatechuic and *p*-coumaric acids) and flavonoids (rutin, quercetin, naringenin, kampferol and derived), are of particular interest since they are effective free radical scavengers through the presence of *para*-hydroxyl groups. These compounds are able to regulate cellular signaling processes during inflammation or may operate as signaling agents themselves thus reducing the risk of neurodegenerative diseases, cancer and aging [7,8]. Flavonoids are the main group of phenolics in tomatoes and include flavonols (such as quercetin), flavanols (such as catechins), flavanones (such as naringenin), anthocyanidins and stilbenes (such as resveratrol). These compounds are usually located in the skin and contribute to the aroma, fragrance and color [9,10]. It has been demonstrated that rutin, quercetin, glycosides of quercetin, resveratrol and catechin are active in rheumatoid arthritis and exert intestinal anti-inflammatory activity. Their mechanisms of action comprise the inhibition of differentiation and function of osteoclast/macrophage and the modulation of estrogen [11,12,13,14]. Diet-derived flavonoids have also been demonstrated to possess antiallergic, antiulcer, antioxidant, antiradical, antidiabetic, cardioprotective, antiviral, antibacterial, antifungal, antiproliferative and anticarcinogenic activities [15]. Food-derived quercetin is able to inhibit the growth of various cancer cell lines, although the exact mechanism of this action is not thoroughly understood [15].

To date, only a few studies have focused on the impact of processing on the general nutritional quality and antioxidant activities of tomato fruits. In addition, little is known regarding the biological activities of tomato polyphenolic antioxidants [16,17]. Since during tomato pasteurization several changes in bioactive molecules composition can occur [18,19], the present study was aimed at investigating the effect of a classical processing procedure on the antioxidant content and in particular on the phenolic profiles of yellow tomatoes. These varieties have been selected on the basis of their high amount of bioactive molecules and, in particular, of phenolic compounds in the fresh fruit [20]. We characterized their nutritional value (including carotenoids, ascorbic acid and phenolics content) before and after processing. In addition, we evaluated their effect, before and after processing, on three human cancer cell lines by using MTT assays.

## 2. Results and Discussion

### 2.1. Content of Antioxidant Compounds 

Herein we evaluated the content of bioactive compounds in yellow tomato genotypes before and after processing. These genotypes are one Italian (GiàGiù, coded E40) and two Bolivian landraces (M-4 and 284, coded E87 and E92, respectively) and all are cherry-type tomatoes. They were previously selected in our laboratory for their high levels of ascorbic acid (AsA) and/or phenolics [20]. Before chemical extraction, the moisture content both in the processed and un-processed samples was measured and found to be around 93% for all the genotypes under study. The solid content for each sample is reported in Table 1.

**Table 1:** Solid content (g/100 g FW), total carotenoids (mg/100 g FW), lycopene (mg/100 g FW) and beta-carotene (mg/100 g FW) content in yellow tomato lines before (NP) and after (P) processing.

Genotype	Solid Content		Total Carotenoids		Lycopene		β-Carotene	
	NP	P	NP	P	NP	P	NP	P
E40	7.20 ± 0.05	7.60 ± 0.06	0.68 ± 0.01	0.66 ± 0.03	n.d	n.d	0.68 ± 0.1	0.42 ± 0.01 **
E87	7.03 ± 0.03	6.75 ± 0.05	0.53 ± 0.1	0.62 ± 0.03	n.d	n.d	0.53 ± 0.1	0.42 ± 0.02
E92	5.61 ± 0.04	6.25 ± 0.07	0.57 ± 0.2	0.93 ± 0.06 **	n.d	n.d	0.57 ± 0.2	0.61 ± 0.03

Total values are presented as means ± SD (*n* = 9). NP: Not processed. P: Processed. Asterisks indicate values that are significantly different from the non-processed samples (** *p* < 0.01).

#### 2.1.1. Carotenoids

The levels of total carotenoids, lycopene and β-carotene before and after processing are reported in Table 1. Total carotenoids in the genotypes E40 and E87 did not reveal a significant change after heat treatment, whereas a significant mean increase was observed in E92 fruits (63.1%). As expected, the analyzed yellow varieties did not show the presence of lycopene in either unprocessed or processed samples as also reported in earlier studies [20,21]. β-Carotene, the major found carotenoid, decreased significantly (*p* < 0.01) after the heat treatment only in the E40 genotype. Conflicting data on tomato carotenoid stability during thermal processing can be found in the literature. Many authors have reported that carotenoid content was stable in tomatoes submitted to different thermal treatments [22,23]. Georgé *et al.* [21] found that in yellow tomato genotypes β-carotene content was greatly reduced in processed yellow tomatoes compared to fresh tomatoes, while the thermal treatment did not significantly affect the amount of β-carotene and lycopene in red tomatoes. On the contrary, Capanoglu *et al.* [18] showed a significant decrease in both lycopene (32%) and β-carotene (36%) content in processed red tomatoes, while other authors reported an increase in lycopene amount in tomato products. In particular, Re *et al.* [24] found a general increase in the lycopene level in several processed products, with up to 30% in a tomato paste. These contrasting results may be due to the temperatures and durations adopted for the different processing methods. 

#### 2.1.2. Ascorbic Acid and Total Phenolics

The levels of ascorbic acid (AsA) in both fresh fruit and processed samples are reported in Table 2. In the three analyzed varieties (E40, E87 and E92), a significant decrease (*p* < 0.001) was observed in the processed samples with respect to the mean value in fresh fruits (59.8%, 54.8% and 48.4%, respectively). Several examples of AsA loss during thermal processing of tomato products have been reported. In accordance with our data, Abushita *et al.* [16] and Capanoglu *et al.* [18] observed a loss of about 50% after thermal processing, whereas Pérez-Conesa *et al.* [25] reported a mean AsA degradation value of 90% after pasteurization of tomato purées. Gahler *et al.* [26] correlated a reduction in AsA levels in different tomato products with an increase in the heating time and the number of processing steps. Dewanto *et al.* [27] found a loss of AsA in heat-processed tomatoes at 88 °C with an estimated *D*_88°C_ value (the time taken for 90% reduction of the initial vitamin C content at 88 °C) of 276 min. The loss of ascorbic acid during processing could be decreased by using mild treatments and lower temperatures [28]. In this study, spectrophometric methods were used to study the effects of processing on phenolic compounds content. In accord with the behavior observed for AsA, total phenolics levels exhibited a general reduction after thermal treatment (Table 2). Mean decreases of 12%, 16.6% and 26% were found in E40, E87 and E92 from the initial mean values of 50.9 ± 1.7, 52.9 ± 1.8 and 53.5 ± 1.2 mg/100 g FW, respectively. The changes in the content of phenolic compounds we observed in this work could be due to the oxidation of the molecules due to the presence of oxidative and hydrolytic enzymes released during the process. Total flavonoids amount did not reveal a significant variation after processing in E40, while a significant increase of 20.5% and 76.9% (*p* < 0.001) was observed in the genotypes E87 and E92, respectively (Table 2). We observed a general increase of the ratio flavonoids/total phenolics in the three analyzed genotypes after processing. In particular, the increase of this parameter after the treatment ranged from 1.15 fold in E40 to 2.45 fold in E92. Marinova *et al.* [29] found a ratio of around 0.17 in red tomato fruits.

Our data are consistent with evidence from the literature. Indeed, Georgé *et al.* [21] found a significant (28%) decrease of total polyphenol content after thermal processing in yellow tomatoes. Pérez-Conesa *et al.* [25] also observed a decrease of these compounds attributed to the pasteurization step. On the contrary, Gahler *et al.* [26] found an increase in the total phenolic content during the processing of tomato juice and tomato sauce. Dewanto *et al.* [27] reported no significant variations in total phenolic compounds after heat treatment. The differences observed in these studies can be due to the different processing methods adopted, together with the different analyzed matrixes and their composition. However, it has to be taken into consideration that total phenolic values are only indicative of the amount of polyphenols in tomatoes, since no single analytical procedure is able to accurately measure the total polyphenol amount. This is due to the structural diversity found among phenolic molecules and the high variation in content depending on the tested matrix [30,31].

**Table 2:** Ascorbic acid, total phenolics, total flavonoids content and flavonoids/phenolics ratio in yellow tomato lines before (NP) and after (P) processing.

Sample	Ascorbic Acid		Total Phenolics		Total Flavonoids		Flavonoids/Phenolics	
	NP	P	NP	P	NP	P	NP	P
E40	54.09 ± 1.12	21.76 ± 0.97 ***	50.86 ± 1.74	44.73 ± 0.63 ***	22.52 ± 0.73	23.03 ± 1.22	0.44	0.51
E87	64.00 ± 0.91	28.93 ± 1.14 ***	52.86 ± 1.76	44.10 ± 1.05 ***	17.69 ± 0.24	21.31 ± 0.12 ***	0.33	0.48
E92	63.84 ± 0.86	32.95 ± 1.27 ***	53.48 ± 1.22	39.54 ± 0.61 ***	13.21 ± 0.24	23.38 ± 0.12 ***	0.24	0.59

Values are presented as means ± SD (*n* = 9). Data are expressed as mg/100 g FW. NP: Not processed. P: Processed. Asterisks indicate values that are significantly different from the non-processed sample (*** *p* < 0.001).

### 2.2. Identification of Individual Phenolic Compounds

**Figure 1:**
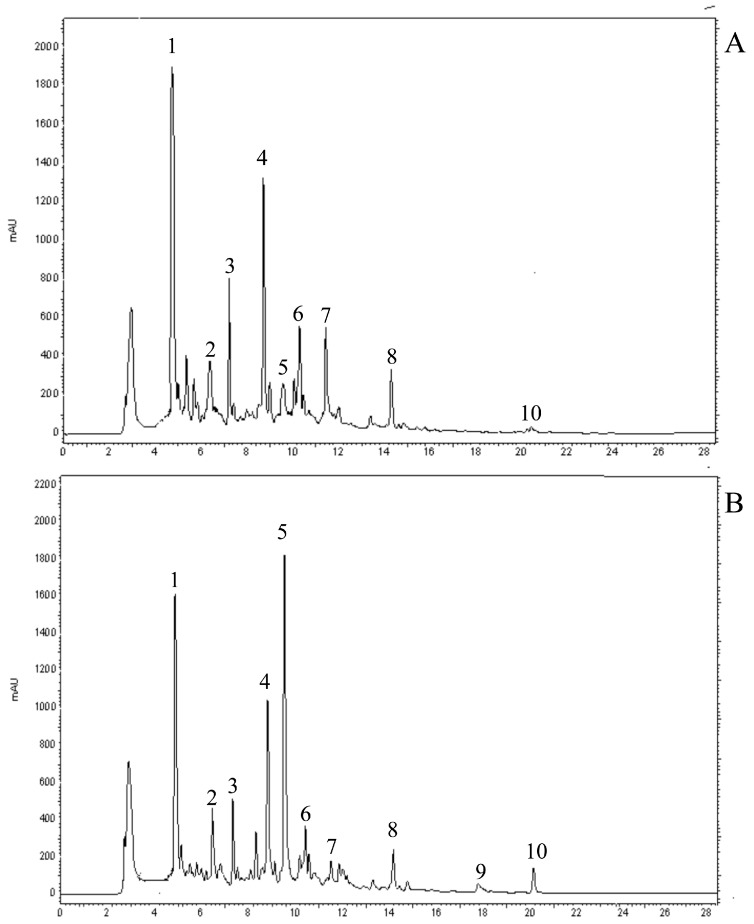
HPLC chromatograms of phenolic compounds in E92 before (**A**) and after (**B**) processing detected at 330 nm. Peaks: 1, chlorogenic acid; 2: caffeic acid; 3, 5-*p*-CQA; 4, rutin; 5, quercetin3-β-D-glucoside; 6, naringin; 7, kampferol-3-rutinoside; 8: chalconaringenin hexoside; 9, quercetin; 10: naringenin and chalconaringenin.

In order to evaluate changes in the composition of individual phenolic compounds after processing, we carried out HPLC analyses (Table 3 and Table 4, Figure 1). Chlorogenic acid, its isomer 5-caffeolylquinic acid (5-*p*-CQA) and rutin were the most abundant compounds in the three analyzed varieties, in both unprocessed and processed forms (Table 3 and Table 4), in accordance with data reported by Garcìa-Valverde *et al.* [6] and Slimestad and Verheul [32].

**Table 3:** Quantification of phenolic acids in yellow tomato lines before (NP) and after (P) processing.

Phenolic acid	Treatment	E40	E87	E92
Chlorogenic Acid	NP	23.16 ± 0.38	15.63 ± 0.63	16.07 ± 0.51
	P	10.10 ± 0.13 ***	8.08 ± 1.21 ***	6.67 ± 0.35 ***
Caffeic Acid	NP	1.80 ± 0.16	1.14 ± 0.11	0.92 ± 0.13
	P	1.41 ± 0.15 **	0.74 ± 0.10 **	0.69 ± 0.08
5-*p*-CQA	NP	7.53 ± 0.71	2.17 ± 0.11	2.92 ± 0.11
	P	4.66 ± 0.30 ***	1.56 ± 0.04 ***	1.69 ± 0.10 ***
Sum of phenolic acids	NP	32.49 ± 1.25	18.94 ± 0.85	19.91 ± 0.75
	P	16.17 ± 0.58 ***	10.38 ± 1.35 ***	9.05 ± 0.53 ***

Values are presented as means ± SD (*n* = 9). Data are expressed as mg/100g FW. NP: Not processed. P: Processed. Asterisks indicate values that are significantly different from the non-processed sample (** *p*< 0.01, *** *p*< 0.001).

**Table 4:** Quantification of flavonoids in yellow tomato lines before (NP) and after (P) processing.

Flavonoid	Treatment	E40	E87	E92
Rutin	NP	29.37 ± 0.76	10.66 ± 0.02	10.78 ± 0.34
	P	21.90 ± 3.78 **	11.26 ± 0.83	8.64 ± 0.61 ***
quercetin	NP	1.17 ± 0.07	0.32 ± 0.08	0.25 ± 0.01
	P	1.55 ± 0.07 ***	0.48 ± 0.04 **	0.75 ± 0.02 ***
Quercetin3-β-D-glucoside	NP	2.34 ± 0.14	1.39 ± 0.21	1.15 ± 0.17
	P	0.96 ± 0.09 ***	0.98 ± 0.06 **	7.42 ± 0.23 ***
Naringin	NP	n.d.	0.22 ± 0.03	0.51 ± 0.02
	P	n.d.	0.20 ± 0.01	0.22 ± 0.03 ***
Naringeninchalcone	NP	0.69 ± 0.13	0.05 ± 0.01	0.08 ± 0.02
	P	n.d.	n.d.	n.d.
Naringenin	NP	n.d.	n.d.	n.d.
	P	1.08 ± 0.05	1.58 ± 0.11	1.84 ± 0.02
Kampferol-3-rutinoside	NP	1.66 ± 0.01	0.44 ± 0.01	0.53 ± 0.01
	P	0.51 ± 0.04 ***	0.23 ± 0.01 ***	0.12 ± 0.01 ***
Chalconaringenin-exoside	NP	0.62 ± 0.09	0.30 ± 0.03	0.39 ± 0.01
	P	0.21 ± 0.02 **	0.22 ± 0.02 **	0.28 ± 0.01 ***
Sum of flavonoids	NP	35.85 ± 1.19	13.38 ± 0.37	13.69 ± 0.58
	P	26.21 ± 4.05 *	14.95 ± 1.07	19.27 ± 0.90 ***

Values are presented as means ± SD (*n* = 9). Data are expressed as mg/100g FW. NP: Not processed. P: Processed. Asterisks indicate values that are significantly different from the non-processed sample (* *p* < 0.05, ** *p* < 0.01, *** *p* < 0.001).

A general significant decrease of phenolic acids was found in the three genotypes after the heat treatment (Table 3). In particular, in the E40 genotype we found that chlorogenic acid, caffeic acid and 5-*p*-CQA were reduced by 56.4%, 21.7% and 38.1%, respectively. For the same compounds, in E87 we found a decrease of 48.3%, 35.1% and 28.1%, respectively. Finally, in E92 decreases of 58.5%, 25% and 42.1%, were found for the three detected phenolic acids. The reduction of chlorogenic acid was also reported by Vallverdú-Queralt *et al.* [19] and Bugianesi *et al.* [33]. Chlorogenic acid and caffeic acid may be oxidized to reactive *o*-quinones through the catalytic oxidation process and so heat treatment could result in their degradation. 

In addition to the loss of total phenolic acids, we found a significant decrease (26.9%) in the content of total flavonoids in E40 (Table 4). However, despite the decrease of phenolic compounds observed, the E40 genotype maintained significantly higher levels of phenolic acids and flavonoids, compared to the other two varieties, even after the thermal treatment (*p* < 0.05). No significant differences were observed in the flavonoid content of E87 extracts, whereas a significant increase in E92 extracts (about 41%) was observed after processing. These last results relate well with those obtained using spectrophometric methods. The increase of flavonoids content in E92 could be also due to the fact that homogenization and thermal processing disrupt cell membranes and cell walls, thus facilitating the release of some nutrients from the tissues, making them more easily extractable from the matrix [34].

In all the analyzed genotypes variations in the distribution of flavonoids were detected after processing. For example, in E40 and in E92 the amount of rutin decreased significantly by about 25.4% and 20%, respectively, whereas in E87 the amount of rutin did not change significantly from the initial value of 10.7 ± 0.02 mg/100 g FW. In accordance with our data, Capanoglu *et al.* [18] found that rutin decreased after treating the samples in a three-effect evaporator unit. Interestingly, we also observed a significant increase in quercetin levels, of about 1.3-, 1.5- and 3-fold in E40, E87 and E92, respectively, and an increase of about 6-folds of quercetin3-β-d-glucoside in E92 from the mean initial value of 1.2 ± 0.17 mg/100 g FW. By contrast, a decrease of quercetin3-β-d-glucoside of 58.9% and 29.5% was found in E40 and E87, respectively. The difference in stability of some phenolics in the three analyzed genotypes could be related to different factors linked to the composition of the analyzed fruits.

Finally, naringenin chalcone was converted into naringenin after heat treatment. This is probably due to unforeseen cyclization of the chalcone to the corresponding flavanone during the processing [35,36]. Though naringenin and naringenin chalcone have analogous chromatographic properties using the HPLC method reported in the Experimental Section, they were easily distinguished due to their different wavelength of their maximum absorptions (280 nm for naringenin and 330 nm for naringenin chalcone).

### 2.3. Antioxidant Activity

In the processed E40 genotype LAA showed a mean increase of 13.2%, from the mean initial value of 39.5 ± 2.5 μmol TE/100g FW in fresh fruit (Table 5). In contrast, E87 revealed a significant decrease after processing (*p* < 0.001). No significant changes were on the other hand observed in the E92 samples. Before processing the genotype E87 showed a significantly higher value of LAA compared with the other two analyzed cultivars (*p* < 0.001), while after processing a significantly higher LAA value was observed in the genotype E40 (*p* < 0.001). These results do not correlate with the amount of total carotenoids and β-carotene calculated in the different samples, and so a significant contribute to the LAA could be attributed to other molecules, such as vitamin E. Indeed, Seybold *et al.* [37] investigated the content of carotenoids and vitamin E in samples of tomato sauce, soup, baked slices, and juice taken after different heating times. The authors demonstrated that carotene amount decreased or was stable, while tocopherol content significantly rose during short-term heating.

**Table 5:** Antioxidant activities in yellow tomato lines before (NP) and after (P) processing evaluated by ABTS and FRAP analyses.

Genotype	LAA		HAA (ABTS)		HAA (FRAP)	
	NP	P	NP	P	NP	P
E40	39.49 ± 2.50	44.70 ± 0.91 *	299.23 ± 4.83	241.28 ± 4.26 ***	387.14 ± 23.71	279.57 ± 3.82 ***
E87	52.25 ± 1.20	30.36 ± 1.44 ***	258.81 ± 6.06	293.50 ± 12.21 **	427.38 ± 11.27	372.86 ± 17.91 *
E92	39.28 ± 0.85	38.69 ± 0.81	308.46 ± 8.22	253.57 ± 11.35 **	493.00 ± 12.56	269.14 ± 11.41 ***

LAA: lipophilic antioxidant activity, HAA: Hydrophilic antioxidant activity. Values are presented as means ± SD (*n* = 9). Data are expressed as μmol TE/100g. NP: Not Processed. P: Processed. Asterisks indicate values that are significantly different from the non-processed sample (* *p* < 0.05, ** *p* < 0.01, *** *p* < 0.001).

HAA was evaluated by both ABTS and FRAP assays. In accordance with the AsA and phenolic content, HAA revealed a general decrease in processed samples. In particular, by ABTS test, decreases of 19.4%, and 17.8% were recorded for E40 and E92, respectively, whereas in E87 an increase of 13.4% was observed. A reduction of 27.8%, 12.7% and 45.4%, was observed by FRAP test for E40, E87 and E92 respectively. The antioxidant potential of tomatoes varied with the assay method used, confirming that the two methods measure different antioxidative effects [38,39]. The discrepancy of the results here obtained by the two different methods was found also in other studies [5,38,39]. In accordance with our results, Graziani *et al.* [22] found a loss of HAA of the samples after heating treatment. Vallverdú-Queralt *et al.* [19] reported an increase in HAA when fresh tomatoes were processed into puree and juices as a consequence of tomato cream addition, whereas the antioxidant level did not increase when processing was performed in the absence of cream. To determine if the measured contents of the analysed samples can be related to antioxidative effects, we calculated the Pearson product-moment correlation coefficients (r). The analyses show that antioxidative data determined by FRAP well correlate with ascorbic acid levels (r = 0.852) and total phenolics (r = 0.885).

### 2.4. Effects of Tomato Extracts on Cell Viability

**Figure 2:**
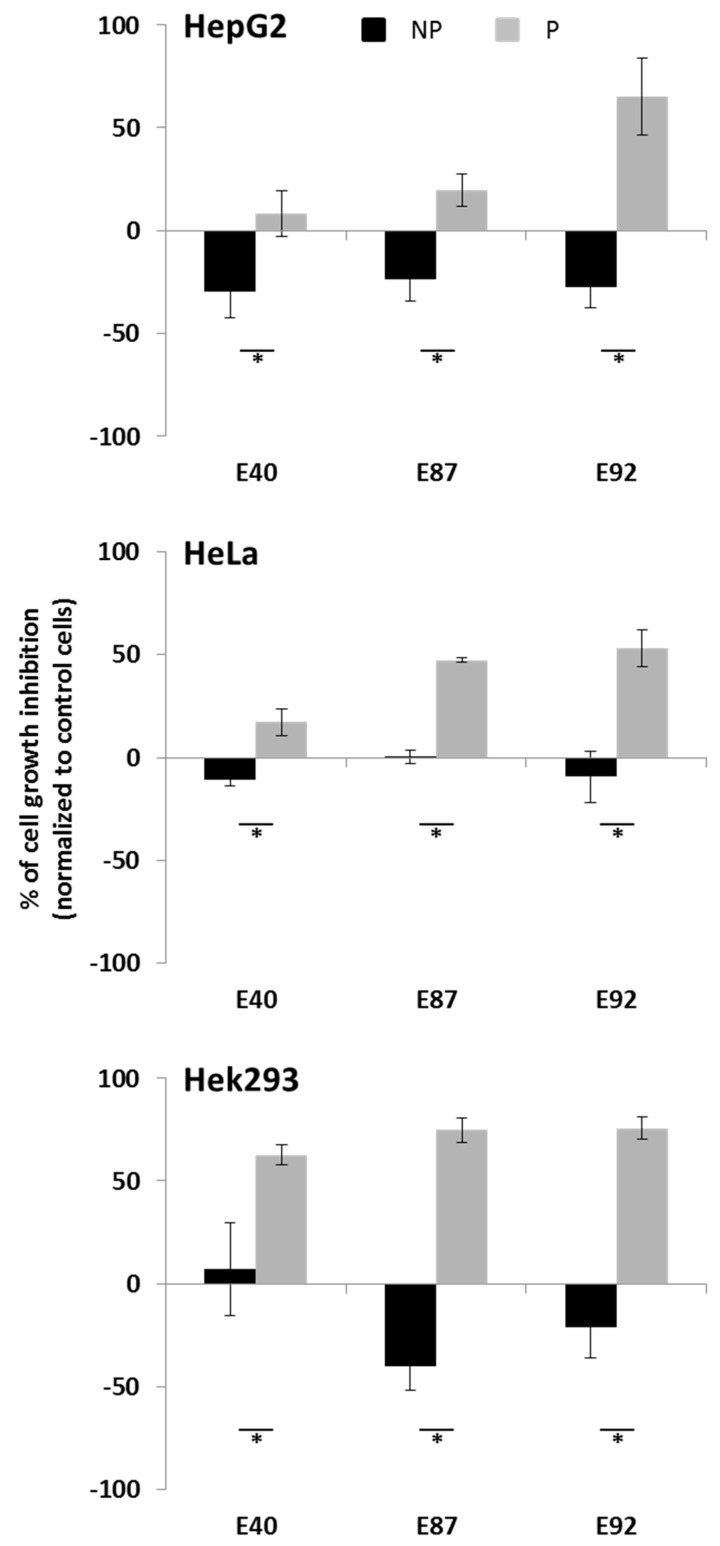
MTT assay of E40, E87 and E92 on human cancer cells. HepG2 (upper panel), HeLa (middle panel) and Hek293 (lower panel), were treated, for 48 h at 37 °C, with 20% (corresponding to a concentration of phenolics ranging between 156 and 212 μg/mL) extracts from E40, E87 and E92 before (black bars) and after (grey bars) heat treatment. All values are given as means ± SD (*n* ≥ 3). Asterisks indicate values that are significantly different from the non-processed sample (* *p* < 0.05).

**Figure A1:**
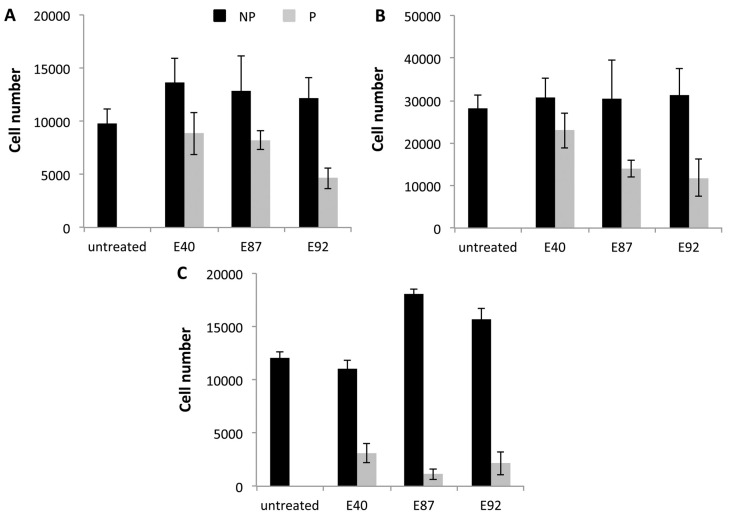
Cell viability assay of E40, E87 and E92 on human cancer cells. HepG2 (**A**); HeLa (**B**) and Hek293 (**C**) were treated for 48 h at 37 °C with 20% (corresponding to a concentration of phenolics ranging between 156 and 212 μg/mL) extracts from E40, E87 and E92 before (black bars) and after (grey bars) heat treatment. Cell number was evaluated by trypan blue dye exclusion assay. All values are given as means ± SD.

To test the anticarcinogenic potential of E40, E87 and E92, before and after processing, we used three human cancer cells derived from: liver (HepG2), kidney (Hek293) and cervix (HeLa). Cells were treated for 48h with 20% of tomato extracts, corresponding to a concentration of phenolics ranging between 156 and 212 μg/mL, and cell viability was tested by the MTT reduction assay, as an indicator of metabolically active cells (Figure 2) and by trypan blue dye exclusion assay (Figure A1). By using the MTT reduction assay we observed after processing (grey bars, Figure 2) a cytotoxic effect of E87 and E92 extracts on the analyzed human cancer cells, whereas E40 extract was highly toxic only on renal cancer cells. No significant effect on cell viability was observed when cells were exposed to the same extracts before processing (black bars, Figure 2). Noteworthy, we observed a general proliferative effect of non-processed tomato extracts on the cell lines analyzed. Since it is known that cancer cells are actively proliferating, the presence of nutrients in combination with low levels of flavonoids may have a positive effect on cell growth [5]. Previous studies demonstrated that processed tomato products inhibited the growth on cancer cells more than the fresh tomatoes [38,39]. The cytotoxic effect of tomato extracts after processing could be due to the increased levels of compounds with reported antiproliferative activity, such as certain flavonoids, which are known to induce apoptosis on different cell lines [40]. In particular, quercetin3-β-d-glucoside has been reported to possess antiproliferative activity on different cell lines [41,42]. The sugar moieties confer to quercetin glycosides a higher stability in water with respect to quercetin. In addition, it has been suggested that the conjugation with glucose enhances quercetin absorption mainly in the small intestine, as this compound can be better absorbed than quercetin and rutin [41]. In addition, it has been demonstrated that quercetin3-β-d-glucoside exerts a more potent antiproliferative effect than quercetin and rutin on various cancer cell lines [41]. You *et al.* [42] demonstrated that the growth-inhibitory effect of the quercetin 3-β-d-glucoside on colon (HT-29 and HCT 116), breast (MCF-7), hepatocellular (HepG2) and lung cancer (A549) cells was higher than that exerted by quercetin and rutin, with rutin being the least cytotoxic. Therefore, the observed E92 cytotoxic effect could be due to the increased quercetin 3-β-d-glucoside content detected after processing only in this genotype (Table 4). It has been reported that quercetin3-β-d-glucoside exhibits a significant antiproliferative activity at doses ranging between 20 and 60 μM, in a dose dependent manner [42,43]. In our study the amount of quercetin 3-β-d-glucoside in the tested tomato extracts was between 8.3 μM and 65 μM. However, we hypothesize that differences in the cytotoxicity of the extracts is due to a “balance” between quercetin 3-β-d-glucoside content and the total phenolic acids levels. Indeed, after thermal treatment, E92 showed the highest flavonoids/phenolics ratio (Table 2). The different antiproliferative activity of E40 extracts compared to the other ones could be also due to the higher level of phenolic acids, in particular chlorogenic acid, recorded in this genotype.

According to this hypothesis, it has been reported that in HepG2 cells chlorogenic acid has no cytotoxic effect and improves cellular tolerance against oxidative factors, by activating survival/proliferation pathways [44]. Finally, the higher cell growth inhibition observed with E87 and E92 tomato extracts after processing, in particular on Hek293 cells, could be due to the presence of higher naringenin levels in these samples. This flavanone is known to induce cytotoxicity and apoptosis in various human cancer cells [45,46,47]. These results have been confirmed by trypan blue dye exclusion assay (Figure A1).

## 3. Experimental Section

### 3.1. Chemicals and Reagents

Standards and reagents were purchased from Sigma (St. Louis, MO, USA), whereas solvents were from Fluka (Buchs, Switzerland). Chromatographic solutions were degassed for 20 min by a Branson 5200 ultrasonic bath (Branson Ultrasonic Corp., Phoenix, AZ, USA).

### 3.2. Plant Material and Processing Material 

Plant material consisted of three tomato genotypes (GiàGiù, 284, M-4) used for fresh consumption. They belong to a wide tomato germplasm collection available at the Department of Agricultural Sciences, University of Naples Federico II, and are referred as E40, E87, E92, respectively. Additional details on the genotypes, including those on their source and distribution, are deposited on the Lab Archives repository hosted http://dx.doi.org/10.6070/H4TT4NXN. Plants were cultivated according to a randomized design with three replicates (10 plants/replicate), in an experimental field located in Acerra (Naples, Italy) in the year 2014. Each sample consisted of 20-pooled fruits per plot. The samples were harvested at full yellow ripe stage, as used in the industry. The fruits were chopped, ground in liquid nitrogen a FRI150 blender (Fimar, Rimini, Italy) to a fine powder, and kept at –80 °C until analyses.

Tomatoes were also processed according to a classical thermal treatment. Briefly, after washing for 5 min with water, tomatoes were treated for 10 min at 92 °C. A part of treated tomatoes was passed through a pulper in order to obtain a puree. Glass cans were filled with 60% of treated whole tomatoes and 40% of puree and were successively vacuum sealed. The filled jars were pasteurized at 100 °C for 60 min and cooled by water. The processed samples were homogenized by using the Fimar FRI150 blender and kept at –80 °C until analyses. Three cans for each genotype were collected and analyzed. The dry matter contents of all the samples was determined by vacuum-drying the samples for at least 12 h at 60 °C to constant weight. The moisture content, both in the processed and un-processed samples, was around 93% for all the genotypes under study. Solid content for each sample is reported in Table 1.

### 3.3. Chemical Extractions

Hydrophilic and lipophilic fractions were obtained according to Rigano *et al.* [5]. Relative to hydrophilic extract, 70% methanol (30 mL) was added to samples of unprocessed or processed tomatoes (3 g) and the mixture was put in an ultrasonic bath for 60 min at 30 °C. Then the mixture was centrifuged at 3500× *g* using a Rotina 420R Hettich 84 Zentrifugen centrifuge (Tuttlingen, Germany) for 10 min at 4 °C, and the supernatant was kept at −20 °C until evaluation of total phenolic compounds, HPLC analysis and hydrophilic antioxidant activity (HAA).

The pellet was extracted three successive times with 16 ml of solution acetone/hexane (40/60, *v*/*v*) using an Ultraturrax 115VAC IKA T 25 High Speed Homogenizer (Cole-Parmer, Vernon Hills, IL, USA) in order to obtain the lipophilic extract. The mixture was centrifuged at 3500× *g* for 5 min at 4 °C according to a modified procedure reported by Zouari *et al.* [48]. The supernatants were collected and stored at –20 °C until the determination of lycopene, β-carotene, total carotenoids and lipophilic antioxidant activity (LAA).

### 3.4. Carotenoid Determination

For carotenoids determination, absorbance of lipophilic extracts was read at 663, 645, 505 and 453 nm. Amounts of β-carotene and lycopene in extracts were calculated according to equations reported by Zouari *et al.* [48]. Total carotenoids were calculated by reading the absorbance at 480, 648, 666 nm according to the formula reported by Wellburn [49]. Results were then converted into mg/100 g FW.

### 3.5. Ascorbic Acid Determination

Ascorbic acid (AsA) determination was performed by a colorimetric method [50] with modifications as reported by Rigano *et al.* [5]. Briefly, TCA 6% (300 μL) was added to sample (500 mg). The mixture was vortexed, incubated for 15 min on ice, and centrifuged at 16,000× *g* for 20 min at 4 °C (Eppendorf Centrifuge 5415R, Hamburg, Germany). Successively, 0.4 M phosphate buffer (20 μL, pH 7.4) and double distilled (dd) H_2_O (10 μL) were added. Eighty milliliters of color reagent prepared as reported by Stevens *et al.* [50] were added. The mixture was incubated at 37 °C for 40 min and the absorbance was read at 525 nm. Three biological replicates were analyzed for each sample. The standard curve was obtained in the range of 0–70 nmol and the values were transformed into mg/100 g FW. 

### 3.6. Total Phenolics and Total Flavonoids Determination 

Total phenolics were determined by the Folin–Ciocalteu assay [51] with modifications reported by Rigano *et al.* [5]. Briefly, Folin-Ciocalteu’s phenol reagent (62.5 μL) and dd H_2_O (250 μL) were added to supernatant (62.5 μL) obtained from the hydrophilic extract obtained as described in Section 3.3. After 6 min, 7% Na_2_CO_3_ solution (625 μL), and dd H_2_O (500 μL) were added to the mixture, which was incubated for 90 min and the absorbance was read at 760 nm. The standard curve was obtained in the range of 0–70 μg/mL gallic acid. Total phenolic content of tomato fruits was expressed as mg gallic acid equivalents (GAE)/100 g FW. Three biological replicates and three technical assays for each biological repetition were analyzed.

Total flavonoids were estimated by the aluminum chloride colorimetric assay reported by Marinova *et al.* [29] with slight modifications. An aliquot (500 μL) of methanolic extract (se Section 3.3) was added to 5% NaNO_2_ (30 μL) and, after an incubation of 5 min, 10% AlCl_3_ (30 μL) was added. After 6 min 1 M NaOH (200 μL) and H_2_O (240 μL) were added and the absorbance of the resulting solution was measured at 510 nm. The standard curve was obtained in the range of 0–100 μg/mL of quercetin. Total flavonoids content was expressed as mg quercetin equivalents (QE)/100 g FW. Three biological replicates and three technical assays for each biological repetition were analyzed.

### 3.7. Individual Phenolics Compounds Determination

Twenty-five millilitres of methanolic extract (Section 3.3) were dried by a rotary evaporator (Buchi R-210, Milan, Italy) and resuspended in 70% methanol (500 μL) containing around 0.175 g of solid weight. The extract was passed through a 0.45 μm Millipore nylon filter (Merck Millipore, Bedford, MA, USA). Flavonoids and phenolic acids were identified and quantified by using a HPLC Spectra System SCM 1000 (Thermo Electron Corporation, San Jose, CA, USA) equipped with a Gemini column (3 μm C18, 110 A, 250 × 4.6 mm; Phenomenex, Torrance, CA, USA) and UV-visible detector (Shimadzu, Riverwood Drive, Columbia, MD, USA) according to the procedure reported by Rigano *et al.* [5]. Chromatograms were recorded at 256 nm for rutin, quercetin and derived, 280 nm for naringenin, 330 nm for chlorogenic acid and derivate, caffeic acid, kampferol-rutinoside, naringenin chalcone and derived. For quantification, integrated peak areas from the tested extracts were compared to the peak areas of known amounts of standard phenolic compounds. The results were expressed as mg/100 g FW. 

### 3.8. Antioxidant Activity Determination

HAA was evaluated using the 2,2′-azinobis-(3-ethylbenzothiazoline-6-sulphonic acid) (ABTS) test and the ferric reducing/antioxidant power (FRAP) method [52]. LAA was evaluated by the ABTS test [53]. 

The ABTS assay is based on the ability of the antioxidants present in the samples to reduce the ABTS^+^ radical action capacity. The FRAP assay displays the reduction of a ferric ion complex to the ferrous form. After the addition of antioxidants, the production of the oxidation products is reduced and the solution changes its color [6,38]. 

An ABTS^•+^ solution was prepared and diluted as described by Miller and Rice-Evans [53]. One hundred microliters of supernatant obtained from the methanolic extraction (Section 3.3) were added to diluted ABTS^•+^ (1 mL) and then the mixture was incubated for 2.5 min. The absorbance was read at 734 nm. The FRAP test was performed by adding acetate buffer (2.5 mL, pH 3.6), TPTZ solution (0.25 mL, 10 mM in 40 mM HCl), FeCl_3_·6H_2_O solution (0.25 mL, 12 mM), and supernatant (150 μL) obtained from the above extraction. After an incubation of 30 min at room temperature, the absorbance of the complex was read at 593 nm. The standard curve resulted linear between 20 and 800 μM Trolox. Results were expressed as micromoles of Trolox equivalents (TE) per 100 g FW.

### 3.9. Cell viability assays

Human adenocarcinoma cells (HeLa), human renal epithelial cells (Hek 293), and human liver hepatocellular cells (HepG2) were from ATCC (American Type Culture Collection, Manassas, VA, USA) and were cultured in Dulbecco’s modified Eagle’s medium (Sigma-Aldrich), supplemented with 10% fetal bovine serum (HyClone, Logan, UT, USA), 2 mM l-glutamine, and antibiotics. Cells were grown in a 5% CO_2_ humidified atmosphere at 37 °C [54]. Cells were seeded in 96-well plates (100 μL/well) at a density of 5 × 10^3^/well. Methanolic tomato extracts, obtained as reported above, were dried by rotovapor (R-210, Buchi, New Castle, DE, USA) at 30 °C, redissolved in dimethyl sulfoxide (DMSO) 5% in PBS, and then added to the cells 24 h after seeding for cytotoxicity assays (20% *v*/*v*). Cell viability was assessed by the MTT assay after 48 h incubation. The MTT reagent, dissolved in DMEM in the absence of phenol red (Sigma-Aldrich), was added to the cells (100 μL/well) to a final concentration of 0.5 mg/mL. Following an incubation of 4 h at 37 °C, the culture medium was removed, and the resulting formazan salts were dissolved by adding isopropanol containing 0.1 N HCl (100 μL/well). Absorbance values of blue formazan were determined at 570 nm using an automatic plate reader (Microbeta Wallac 1420, PerkinElmer, Shelton, CT, USA). The decrease in absorbance in the assay measures the extent of decrease in the number of viable cells following exposure to the test substances calculated by using the following formula: % inhibition of cells = (A control – A test substance)/A control × 100. Three separate analyses were carried out with each extract. Control experiments were performed either by growing cells in the absence of the extract and by supplementing the cell cultures with identical volumes of extract buffer (5% DMSO in PBS). The method used avoids any possibility of a DMSO effect on the results. In parallel experiments, cell viability was evaluated by trypan blue dye exclusion assay. After incubation for 48 h at 37 °C with tomato extracts, cells were detached by trypsin, centrifuged at 1000× *g* for 5 min at r.t. and the cell pellet was resuspended in 0.4% trypan blue buffer (Sigma-Aldrich) and counted in the hemocytometric chamber (Burker chamber, Sigma-Aldrich).

### 3.10. Statistical Analyses

The biological replicates of samples were analysed in triplicate. Quantitative parameters were expressed as the mean value ± SD. Differences among not processed and processed samples were determined by using SPSS (Statistical Package for Social Sciences) Package 6, version 15.0 (SSPS Inc., Chicago, IL, USA). Significance was determined by Student’s *t*-test at a significance level of 0.05. SPSS Package 6, version 15.0 was also used to calculate Pearson’s correlation between measured parameters. The percentage of the variations of quantitative parameters before and after the processing was calculated by using the following formula:

(1) $$\text{Increase or Decrease}\;\left(\%\right)=\frac{\text{value after processing}–\text{ value before processing}}{\text{value before processing}}\times 100$$

## 4. Conclusions

In all the samples analyzed, a decrease in the amount of ascorbic acid, total phenolics, phenolic acids and hydrophilic antioxidant activity was observed after heat treatment. Interestingly, processing was able to enrich tomato fruits in bioactive compounds, such as certain flavonoids. In particular, in the two Bolivian genotypes (E87 and E92), heat treatment led to a significant increase in the level of naringenin and quercetin glycoside, which correlates well with the observed antiproliferative activity of these tomato extracts against some human cancer cells. It is worth saying that the anticarcinogenic potential of foods containing phenolics cannot be based only on the effects of individual compounds but may involve a synergistic effect among phytochemicals [43]. In the Italian E40 genotype, high levels of phenolic acids and flavonoids were detected both in the fresh and processed fruits, and the content of these bioactive compounds indicates that it has a potential use for the production of tomato-based functional food. 

Nowadays, there is an increasing demand of natural products, which are expected to be safe and health-promoting. In this context, our data could have a significant impact on consumer’s food selection, increasing their awareness of the health benefits of fresh and processed tomato fruits.
